# Characteristic Analysis of Trigonelline Contained in *Raphanus sativus* Cv. Sakurajima Daikon and Results from the First Trial Examining Its Vasodilator Properties in Humans

**DOI:** 10.3390/nu12061872

**Published:** 2020-06-23

**Authors:** Maho Sasaki, Yuri Nonoshita, Takashi Kajiya, Nobuhiko Atsuchi, Megumi Kido, Djong-Chi Chu, Lekh Raj Juneja, Yuji Minami, Katsuko Kajiya

**Affiliations:** 1 Graduate School of Agriculture, Forestry and Fisheries, Kagoshima University, Kagoshima 890-0065, Japan; k7451140@kadai.jp (M.S.); minami@chem.agri.kagoshima-u.ac.jp (Y.M.); 2 Department of Food Science & Biotechnology, Faculty of Agriculture, Kagoshima University, Kagoshima 890-0065, Japan; k2644727@kadai.jp; 3 Department of Cardiology, Tenyoukai Central Hospital, Kagoshima 892-0822, Japan; t_kajiya@hotmail.com (T.K.); n-atsuchi@tenyoukai.org (N.A.); 4 Department of Human Life and Science, Kagoshima Women’s College, Kagoshima 890-8565, Japan; kido@jkajyo.ac.jp; 5 ROHTO Pharmaceutical Co., Ltd., Osaka 544-8666, Japan; shu@rohto.co.jp (D.-C.C.); juneja@rohto.co.jp (L.R.J.); 6 Laboratory of Biochemistry & Nutritional Chemistry, The United Graduate School of Agricultural Sciences, Kagoshima University, 1-21-24 Korimoto, Kagoshima 890-0065, Japan

**Keywords:** clinical trial, humans, radish, trigonelline, vascular function

## Abstract

Vascular disease poses a major public health problem worldwide. Trigonelline isolated from *Raphanus sativus* cv. Sakurajima Daikon (Sakurajima radish) induces nitric oxide production from vascular endothelial cells and enhances vascular function. Here, we investigated the characteristics of trigonelline and its effects on endothelial function after consumption of Sakurajima radish by humans. Our results show that Sakurajima radish contains approximately 60 times more trigonelline than other radishes and squashes. Additionally, no significant differences were observed between varieties of Sakurajima radish, suggesting that any type of Sakurajima radish can be ingested for trigonelline supplementation. The effects of cooking and processing Sakurajima radish were also evaluated, as were the effects of freezing, and changes in osmotic pressure and pH. A first-in-human trial using Sakurajima radish showed that ingestion of 170 g/day of Sakurajima radish for ten days increased blood trigonelline concentrations and significantly improved flow-mediated dilation, which is a measure of vascular endothelial function. Overall, our findings suggest that the trigonelline contained in Sakurajima radish may contribute to improved human vascular endothelial function. Hence, Sakurajima radish may enhance vascular endothelial function as a functional food.

## 1. Introduction

Cerebrovascular diseases, such as stroke, and heart diseases, including angina pectoris and myocardial infarction, are the leading causes of death worldwide. Even for patients who recover, a major economic burden is placed on both the patient and society, as patients must undergo long-term treatments and extended bed rest. Hence, there is a need to improve the vascular function of patients in an effort to prevent vascular diseases. Accordingly, our laboratory has been studying the effects of food materials on vascular function and attempting to elucidate the mechanisms through which functional foods may prevent vascular disease [1,2,3].

**Figure 1:**
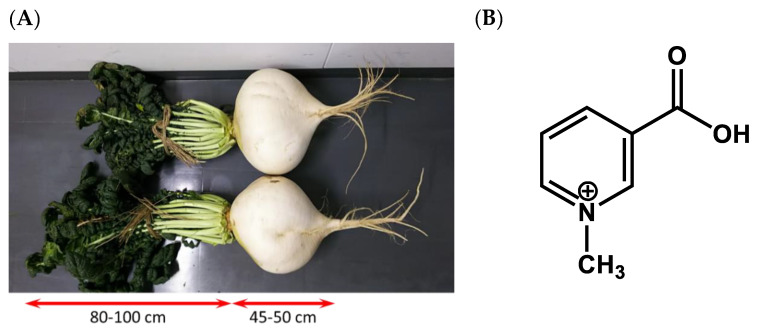
Sakurajima radish (**A**) and chemical structure of trigonelline (**B**).

Blood vessels consist of three layers: the outermost tunica adventitia, the tunica media, and the innermost tunica intima, which contains vascular endothelial cells (VECs). Nitric oxide (NO) is released from VECs to protect blood vessels by regulating their contraction and relaxation and by preventing thrombus formation caused by attachment of white blood cells, and other blood components, to the vascular endothelium [4,5]. Meanwhile, if VECs become damaged by oxidative stress induced by reactive oxygen species, or oxidised low-density lipoprotein (LDL), the production of NO is suppressed, thereby increasing the risk of cardiovascular diseases [6]. Accordingly, improving NO production by VECs is critical for protecting blood vessels. In our previous research, *Raphanus sativus* cv. Sakurajima Daikon (Sakurajima radish; Figure 1A) extracts were found to induce NO production, and its active ingredient was identified as trigonelline (Figure 1B) [1]. Sakurajima radish, produced in Kagoshima, Japan, is famously known as the world’s largest radish cultivar [7,8]. However, no studies have yet evaluated the concentration of trigonelline that becomes absorbed and subsequently transferred into the blood following human consumption of Sakurajima radish. Moreover, although the bioregulatory functions of trigonelline, including improved hypertension, diabetes, and central nervous system disease, have been reported [9,10], the effects of Sakurajima radish on human endothelial function have not been characterised.

Therefore, in this study, we evaluated trigonelline contents in various plants, including several varieties of Sakurajima radish, including ‘Sakurajima Ogojo’, which is a smaller F1 variety with a high germination rate cultivated in Kagoshima Prefecture; the ‘Native farm species’, which is a larger variety with a germination rate of approximately 75% [11,12]; and ‘Others’, which represents other varieties obtained from the markets. Furthermore, we assessed the effects of cooking and processing Sakurajima radish on trigonelline and conducted the first trial of Sakurajima radish in humans.

## 2. Materials and Methods 

### 2.1. Materials

Sakurajima radish, Aokubi radish (*Raphanus sativus var. Longipinnatus*), coffee cherry (*Coffea arabica*), and squash (*Cucurbita maxima*), cultivated in Kagoshima, Japan, were used in this study. Different varieties of Sakurajima radish were obtained, including ‘Sakurajima Ogojo’, an F1 variety with lower occurrence of hollow cavity and pores; ‘Native farm species’, are the largest varieties and have been inherited for many years; and ‘Others’ which represented a mixture that do not classified other varieties. Seeds of Sakurajima radish were obtained from the Japan Agricultural Cooperative (Kagoshima, Japan). Porcine VECs were purchased from Cosmo Bio Co., Ltd. (Tokyo, Japan). Trigonelline and methanol for high-performance liquid chromatography (HPLC) were purchased from FUJIFILM Wako Chemical Corporation (Osaka, Japan).

### 2.2. Quantification of Trigonelline

The roots and leaves were separated and cut into small pieces. The roots were processed using a homogeniser and lyophilised to generate powdered raw material. One mL of methanol/H_2_O/acetic acid solvent (95.0%/9.5%/0.5%, *v*/*v*/*v*) was added to 25 mg raw material and mixed in a vortexer, followed by 5 min of ultrasonic treatment. The sample was centrifuged twice at 1600× *g* for 10 min at 4 °C (cooling centrifuge 3500; KUBOTA Corporation Co., Ltd., Tokyo, Japan); the supernatant was then collected and concentrated by drying. The dry sample material was weighed and dissolved in an appropriate solvent prior to use in experiments.

After filtration through a 0.45-μm filter (Toyo Roshi Kaisha, Tokyo, Japan), the samples were analysed by HPLC with an ultraviolet-visible adsorption detector and a photodiode array detector (Extrema, Jasco, Tokyo, Japan). The conditions for HPLC analysis were as follows: the C18 reversed-phase column (COSMOSIL 5C_18_-AR-300, 5 μm, 4.6 mm I.D., 250 mm; NACALAI TESQUE, INC., Kyoto, Japan) was maintained at 40 °C, and detection was conducted at 265 nm. The mobile phase consisted of 10 mM phosphoric acid solution (A) and methanol (B). We used a gradient of 0 min with 10% solution B, and 0–10 min with a direct increase in solution B of up to 40%. The flow rate was 0.7 mL/min, and the injection volume was 10 μL.

### 2.3. Cooking and Processing

After harvesting Sakurajima radish, 1 g of the washed product was weighed to obtain a raw sample. The samples used for analysis of the effects of boiling were sliced thinly and boiled at 100 °C for 1, 10, 20, and 30 min; whereas the samples used for analysis of the effects of high-temperature cooking were baked in an oven or fried in oil for 1, 5, 10, 15, and 20 min at 180 °C. The samples used for analysis of the effects of freezing were stored in a deep freezer at −80 °C after initial freezing in liquid nitrogen. In addition, salted samples were immersed in 5% salt for 24 h, and pickled samples were boiled with vinegar (1 mL), water (1 mL), sugar (0.2 g), and salt (0.06 g; pH 3.8) for 24 h.

### 2.4. First Trial of Sakurajima Radish Examining the Vasodilator Property in Humans

Fourteen healthy volunteers (seven men and seven women, age 33.9 ± 6.7 years) participated in this study. Sakurajima radish (native species) was consumed at 170 g/day (trigonelline content: 61.2 mg), which was assumed to be sufficient for promoting NO production from VECs [1]. Other lifestyle factors were not altered. The intake period was ten consecutive days, and participants were permitted to either eat the entire 170-g portion, which was packaged using a vacuum packer (MINI JUMBO, RO18765-1; Nichiwa Electric Corporation, Tokyo, Japan), at once or divide it into multiple servings. Additionally, there were no limitations to the method used to cook the radish. Blood pressure (BP), pulse, and weight were measured before consumption and then again after ten days of consumption of Sakurajima radish. Blood samples were collected before consumption and then again after ten days of consumption of Sakurajima radish, and general biochemical tests (white blood cell [WBC]; haemoglobin [Hb]; platelet [Plt]; LDL cholesterol [LDL-C]; high-density lipoprotein cholesterol [HDL-C]; triglyceride [TG]; fasting plasma glucose [FPG]; uric acid [UA]; blood urea nitrogen [BUN]; creatinine [Cr]; sodium [Na]; potassium [K]; chloride [Cl]; aspartate transaminase [AST]; and alanine transaminase [ALT]) were performed. For analysis of vascular endothelial function, flow-mediated dilation (FMD) values were determined using an ultrasonic diagnostic imaging device (UNEXEF18VG; UNEX Co., Nagoya, Japan), which is an automated edge detection system for measurement of brachial artery diameter. FMD represents endothelium-dependent, largely NO-mediated, dilatation of conduit arteries in response to an imposed increase in blood flow and shear stress and is a tool for examining the pathophysiology of cardiovascular diseases to potentially identify individuals who are at increased risk of future cardiovascular events [13]. FMD was automatically calculated as the percentage change in peak vessel diameter from the baseline value. Percentage of FMD [(peak diameter − baseline diameter)/baseline diameter] was used for analysis [14]. Impaired endothelial function plays an important role in the initiation of atherosclerosis, and brachial FMD is a predictor of coronary artery disease severity [15]. To evaluate arteriosclerosis, brachial-ankle pulse wave velocity (baPWV) values were examined using a BP pulse wave tester (BP-203RPE III; Omron Healthcare, Kyoto, Japan). The blood concentration of trigonelline was determined by centrifugation (1000× *g*, 5 min) of plasma, and values were then quantified by HPLC as described above. This test was conducted with the approval of the institutional Ethics Review Committee by an outside expert (approval no. 31-1), and written informed consent was obtained from all participants.

### 2.5. Statistical Analyses

Significant differences among groups were assessed using Student’s *t*-tests. Data are represented as means ± standard deviations (SDs). Results with *p* values < 0.05 were considered statistically significant. In the human trial, discrete data were presented as frequencies and percentages, and continuous variables were presented as means ± SDs. Continuous variables were compared using either *t*-tests or Mann Whitney U tests, and results with *p* values < 0.05 were considered statistically significant.

## 3. Results

### 3.1. Comparison of Trigonelline Content in Sakurajima Radish

Of the approximately 300 types of plants investigated, those in which trigonelline was detected were radish, coffee, and squash. When the amount of trigonelline in the root extracts of Sakurajima radish was measured and converted per weight of raw Sakurajima radish, raw Sakurajima radish was found to contain 360 ng/mg trigonelline. Setting this value as 100%, Table 1 shows the relative values of trigonelline contents in other plants. Aokubi radish contained only 1.75% the trigonelline content as Sakurajima radish, and the same amount as squash. In contrast, fresh coffee cherry contained approximately 81.7% of the trigonelline as Sakurajima radish. However, the source of coffee that is often consumed does not originate from coffee cherry, but rather the seeds obtained by removing fermentable skin and pulp from coffee cherry. In addition, coffee cherry seeds cannot be eaten in their natural form, but instead by first be roasted at high temperatures. The trigonelline value decreased to 17.15% after roasting at 185 °C for 15 min. After extraction with hot water at 100 °C (French press method), coffee cherry lost up to 0.01% of its trigonelline content. Hence, the amount of trigonelline consumed from Sakurajima radish is overwhelmingly high.

### 3.2. Differences in Trigonelline Content between Sakurajima Radish Varieties

**Figure 2:**
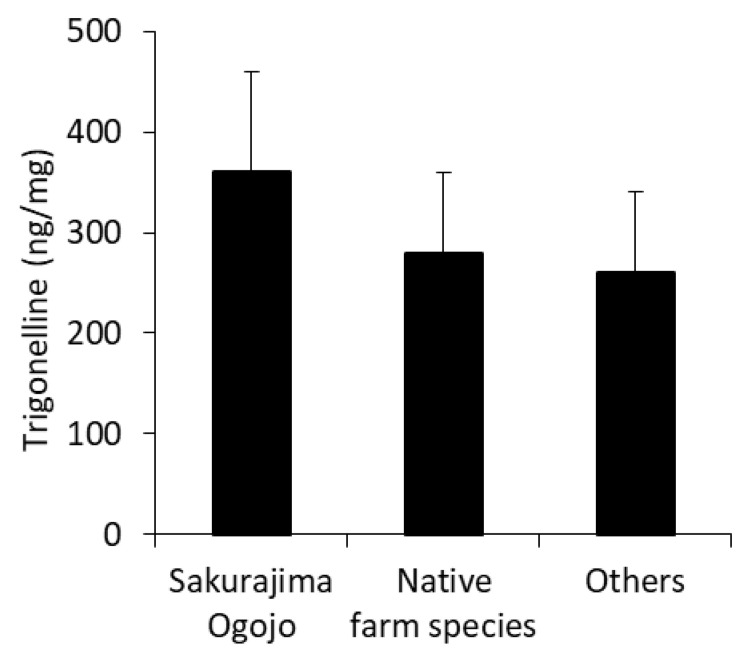
Differences in trigonelline contents among Sakurajima radish varieties. ‘Sakurajima Ogojo’ are an F1 variety with lower occurrence of hollow cavity and pores. ‘Native farm species’ are the largest varieties and have been inherited for many years. ‘Others’ which represented a mixture that do not classified other varieties. All values are represented as means ± SD. There were no significant differences observed between these varieties.

The average trigonelline content in Sakurajima radishes was 260–360 ng/mg, which was significantly higher than that of other plants. However, although there was a tendency for the amount of trigonelline in “Sakurajima Ogojo” to be higher than that in the other varieties, no significant differences were observed as each variety showed large individual differences (Figure 2). These findings suggest that all varieties of Sakurajima radish contained high amounts of trigonelline.

### 3.3. Effects of Cooking and Processing Sakurajima Radishes on Trigonelline Contents

**Figure 3:**
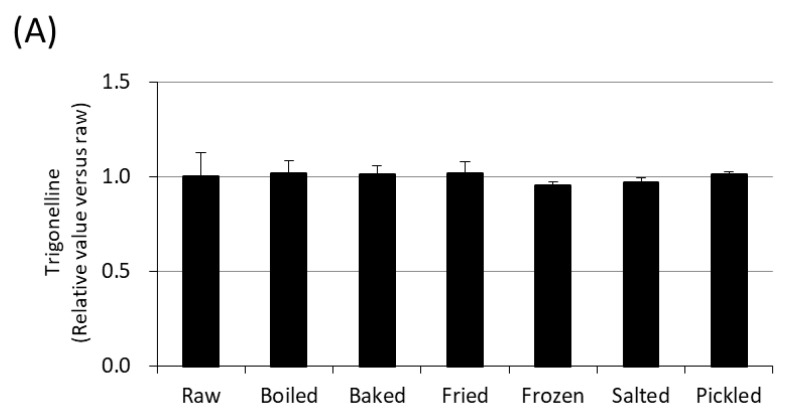

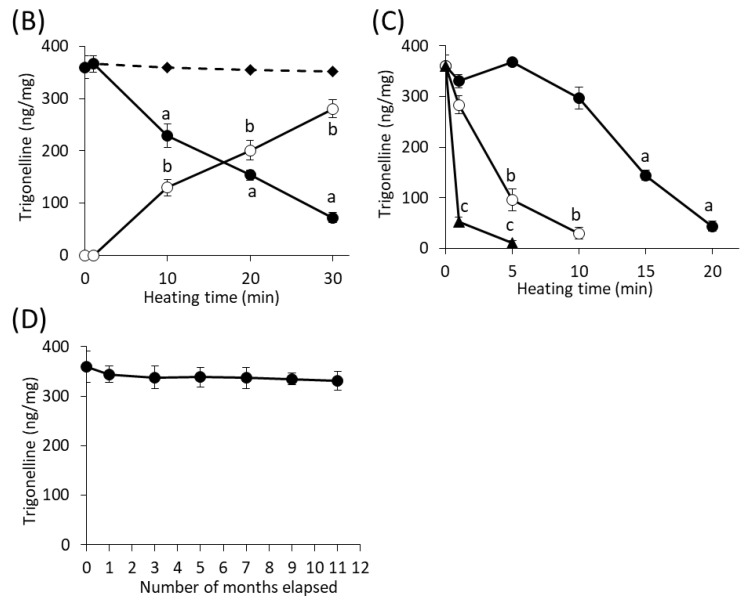
(**A**) Effects of cooking and processing on trigonelline content. (**B**) Time-dependent changes in trigonelline content in radish (black circles), infusion (white circles), and toral (broken line) after heating in hot water at 100 °C. (**C**) Changes in trigonelline contents after heating in an oven at 180 °C (black circles), 185 °C (white circles), or 190 °C (black triangles). (**D**) Time course of changes in trigonelline contents when stored in a freezer at −30 °C. All values are represented as means ± SD. For each time point, means with a different letter are significantly different, *p* < 0.05.

Figure 3A shows the effects of cooking and processing radishes on trigonelline content, with the amount of trigonelline contained in raw Sakurajima radish set at 1. Although trigonelline is a water-soluble substance, all of it was detected in the radish when boiled for approximately 1 min. After boiling for more than 10 min, the amount of trigonelline was reduced to approximately half, and trigonelline was detected in the infusion. Since no change was observed in the total amount, it is considered that trigonelline was not decomposed by heating at 100 °C but rather was discharged from the radish to the surrounding infusion (Figure 3B). After high temperature cooking, the centre temperature of the Sakurajima radish reached 180 °C. Heating at 180 °C for 10 min did not affect trigonelline, however, after 15 min it was found to decompose. Meanwhile, at 185 °C, a small amount of trigonelline was decomposed even after heating for 1 min; and after 10 min only very low levels were retained in the radish. At 195 °C, it decomposed rapidly after heating for only 1 min, and after 5 min it was barely detectable (Figure 3C). Furthermore, the level of trigonelline was reduced by 5% in Sakurajima radish after one month of freezing, and was suppressed by only 8% after 11 months (Figure 3D). Almost all trigonelline was detected in the radish regardless of changes in osmotic pressure due to salt and in pH due to vinegar. These findings also demonstrate that trigonelline was stable for cooking and processing Sakurajima radish.

### 3.4. First Trial of Sakurajima Radish Examining the Vasodilator Property in Humans

Following the first trial of Sakurajima radish in humans, no changes were observed in BP, or body weight (Table 2). 

**Figure 4:**
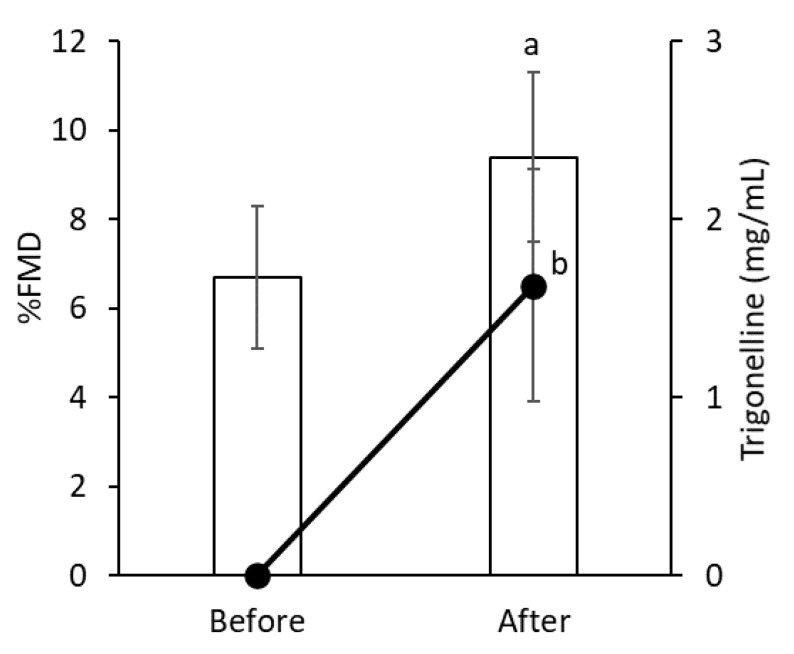
FMD levels (bar graph) and trigonelline concentrations (line graph) before and after consumption of Sakurajima radish for ten days. All values are represented as means ± SD. The means with a different letter are significantly different (a, difference in FMD level; b, difference in trigonelline concentration; *p* < 0.05 versus before).

However, a trend towards reduced pulse was found after ten days (73.6 ± 11.0/min versus 70.9 ± 11.7/min, *p* = 0.07), which was not clinically significant. Moreover, biochemical examination of blood samples following ingestion of Sakurajima radish for ten days showed a significant decrease only in TG (110.1 ± 123.4 mg/dL versus 60.8 ± 33.0 mg/dL, *p* = 0.04; Table 3). Meanwhile, platelet counts were only slightly decreased (27.2 ± 5.9 × 10^4^/μL versus 26.1 ± 5.8 × 10^4^/μL, *p* = 0.10), which was not clinically significant. Furthermore, although trigonelline was not detected in any of the blood samples before consumption of Sakurajima radish, 1.6 ± 0.2 mg/mL trigonelline was detected after ten days of consumption (Figure 4). This indicates that trigonelline was absorbed following intake of Sakurajima radishes. In addition, the %FMD, which is normal above 6.0, was significantly improved (6.7 ± 1.6% versus 9.4 ± 1.9%, respectively; *p* = 0.0016) following consumption for ten days. Lastly, baseline brachial artery diameter was 3.5 ± 0.5 mm before and 3.4 ± 0.6 mm after ten days consumption (*p* = 1.0). Hence, the baPWV did not show any significant differences (1184.4 ± 201.4 versus 1179.8 ± 197.8, respectively; *p* = 0.4). 

## 4. Discussion

Following comparison of the amount of trigonelline within the tested food ingredients, we found that Sakurajima radish served as the most significant source. In fact, the Aokubi radish, which belongs to the same Japanese radish family, contains only approximately 1/57 of the trigonelline content of Sakurajima radish. These results support those previously reported, which indicated a high NO-producing capacity of Sakurajima radishes [1]. Moreover, coffee products that are commonly consumed may contain low levels of trigonelline, unless prepared via specific manufacturing methods that do not require roasting [16]. Although the biosynthetic pathway of trigonelline has not been fully elucidated, the production of trigonelline from water-soluble vitamin nicotinic acid is likely catalysed by *N*-methyltransferase [17,18,19]. Considering that many plants use nicotinamide and nicotinic acid as final products, it is postulated that Sakurajima radish and coffee cherry synthesise the final product trigonelline via nicotinamide and nicotinic acid as intermediate products by activation of trigonelline synthase.

The effects on trigonelline following cooking and processing were also examined, and it was shown that trigonelline is highly stable even as a water-soluble substance. In fact, after boiling Sakurajima radish at 100 °C, trigonelline was detected in the radish for approximately 1 min, meanwhile, the amount of trigonelline was reduced to approximately half, and detected in the infusion after boiling for 10 min. In Japanese cuisine, Sakurajima radish is also commonly baked in the oven or fried in oil, hence, the effects of high temperature cooking were also investigated. Interestingly, following 10 min of baking or frying the trigonelline at 180 °C remained within Sakurajima radish rather than becoming exuded, as was observed following boiling. Furthermore, trigonelline was found to decompose when exposed to temperatures of 180 °C for more than 15 min, or temperatures ≥ 185 °C for any amount of time. This is reasonable considering the trigonelline reduction results obtained following roasting coffee shown in Table 1. 

In addition, unlike Aokubi, which can be cultivated in approximately 3 months, Sakurajima radish requires a cultivation period of 5 to 6 months, with a harvest period of only 3 months per year. Hence, effective preservation techniques must be employed to allow for the distribution of Sakurajima radish throughout the entire year. Freezing, may be one such method as retention of trigonelline in frozen Sakurajima radish was very high (95%; Figure 3A), and was found to be stable over an 11 months period. Taken together, these results suggest that Sakurajima radish can be subjected to general cooking and processing methods while retain high trigonelline contents, consistent with the many types of Sakurajima radish (pickles, sprinkles, rice crackers, Japanese sweets, frozen sweets, jelly drinks, and chips) found in the market.

The first trial of Sakurajima radish examining the vasodilator property in human found no significant differences in the blood biochemical parameters between the two groups i.e., those that consumed radish and those that did not, save for TG values. TG-rich lipoproteins represent causal risk factors for atherosclerotic cardiovascular diseases, and all-cause mortality [20]. A previous study suggested that trigonelline might reduce triglyceride levels, which was supported by our data [21]. Trigonelline was not detected in any blood samples before consumption of Sakurajima radish; meanwhile significant levels of trigonelline were detected after consumption of Sakurajima radish for ten days. This indicates that trigonelline was absorbed following consumption of Sakurajima radish. Furthermore, FMD is a surrogate marker of endothelial function that can complement clinical symptoms of structural arterial disease, while facilitating early diagnosis and prediction of cardiovascular diseases outcomes. The %FMD, which is considered normal above 6.0, was significantly improved in individuals who consumed Sakurajima radish for ten days compared to those that did not (9.4% ± 1.9% versus 6.7% ± 1.6%, respectively; *p* = 0.0016). Meanwhile, the baPWV did not exhibit significant differences between the two groups. Although endothelial dysfunction contributes to progression of structural arterial stiffness, consumption of Sakurajima radish for ten days does not appear to be sufficient to affect baPWV. Hence, longer periods of ingestion may be required to significantly affect baPWV and contribute to the prevention of atherosclerosis.

Recently, trigonelline contained in coffee was reported to have various bioregulatory functions in cells and animal models, including hepatic lipid accumulation, lipotoxicity [22], suppression of diabetes mellitus [23], and suppression of diabetic nephropathy [24]. In addition, human studies have reported the effects of consuming coffee containing trigonelline, including acute effects on glucose tolerance [25], management of patients with Parkinson’s disease [26], and reduced levels of spontaneous DNA strand breaks in WBCs [27]. However, coffee also contains abundant amounts of caffeine, which has a stimulating effect, and during roasting, coffee metabolites undergo complex Maillard reactions, producing melanoidins and other degradation products; thus, the results are often difficult to interpret. Nevertheless, herein we found that the trigonelline contained in Sakurajima radish likely has the capacity to directly contribute to improved human vascular endothelial function. Kuroda et al. found that the underlying mechanism for stimulating NO production by trigonelline, an active constituent of Sakurajima radish involves endothelial NO synthase activation by the phosphorylation of Ser1177 and the dephosphorylation of Thr495, which is triggered by elevated concentrations of cytoplasmic Ca^2+^, resulting from the activation of Ca^2+^ channels in VECs [1]. The molecular mechanisms of vasodilation in human by Sakurajima radish in this study appear consistent with those reported by Kuroda et al. 

## 5. Conclusions

In this study, we compared the trigonelline contents of various plants, including Sakurajima radish varieties. Importantly, we found that Sakurajima radish contained approximately 60 times more trigonelline than Aokubi radish or squash. Furthermore, fresh coffee cherries, which contained the second highest amount of trigonelline, showed reduced trigonelline contents after roasting, suggesting that the coffee generally consumed by humans may be low in trigonelline. Therefore, Sakurajima radish was identified as the most suitable source of trigonelline. Moreover, although there were large individual variations among varieties of Sakurajima radish, the differences were not significant, suggesting that all types of Sakurajima radish likely contained high amounts of trigonelline. Evaluation of the effects of cooking and processing revealed that trigonelline was relatively resistant to heating at 100 °C for approximately 1 min; however, trigonelline contents in radish decreased rapidly as the elapsed time increased and detected in the infusion. Additionally, trigonelline was decomposed after heating for 10 min at 180 °C or 1 min at 185 °C and 190 °C. In contrast, the compound was quite stable after freezing for up to 11 months. Based on these results, we conducted the first trial of Sakurajima radish consumption in humans. Ingestion of 170 g/day Sakurajima radish for ten days effectively increased blood trigonelline concentrations and significantly improved %FMD, which represents vascular endothelial function. Overall, our findings revealed that trigonelline contained in Sakurajima radish is likely to directly contribute to the improvement of vascular endothelial function in humans. Thus, Sakurajima radish may have applications as a functional food for enhanced vascular endothelial function. Despite the strengths and positive results obtained in this study, we note that the efficacy of Sakurajima radish was only investigated in healthy Japanese volunteers. Thus, further large-scale clinical trials in patients with vascular disease are needed to clearly establish the clinical benefits of Sakurajima radish.

## Figures and Tables

**Table 1:** Comparison of trigonelline amounts in different products.

Plant	Type	Relative Value (%)
Radish	Sakurajima	100
	Aokubi	1.75
Coffee	Fruit	81.74
	Roast	17.15
	Extraction	0.01
Buttercup	Seeds	1.50
Squash	Fruit	1.00

**Table 2:** Changes in blood pressure (BP), pulse, and body weight.

		Before	After	*p* Value
Systolic BP	mmHg	120 ± 13.6	115.8 ± 10.8	0.72
Diastolic BP	mmHg	70.8 ± 11.0	68.2 ± 10.7	0.66
Pulse	/min	73.6 ± 13.7	70.9 ± 11.7	0.07
Body weight	kg	58.1 ± 9.4	58.0 ± 9.4	1.0

**Table 3:** General biochemical examination of blood collected from participants.

		Before	After	*p* Value
WBC	/μL	5785.7 ± 1287.8	6228.6 ± 1658.1	0.12
Hb	g/dL	14.6 ± 1.6	14.5 ± 1.5	0.58
Plt	×10^4^/μL	27.2 ± 5.9	26.1 ± 5.8	0.10
LDL-C	mg/dL	108.5 ± 21.1	113.6 ± 29.0	0.33
HDL-C	mg/dL	79.5 ± 18.3	77.7 ± 14.9	0.47
TG	mg/dL	110.1 ± 123.4	60.8 ± 33.0	0.04
FPG	mg/dL	87.5 ± 4.4	90.4 ± 7.1	0.08
UA	mg/dL	4.4 ± 1.5	4.9 ± 1.6	0.23
BUN	mg/dL	13.4 ± 3.0	12.6 ± 2.8	0.24
Cr	mg/dL	0.7 ± 0.2	0.7 ± 0.2	0.71
Na	mEq/L	142.0 ± 1.4	140.6 ± 1.3	1.00
K	mEq/L	4.2 ± 0.3	4.2 ± 0.2	1.00
Cl	mEq/L	101.6 ± 1.4	103.1 ± 1.0	1.00
AST	IU/L	20.5 ± 4.7	20.3 ± 4.2	0.89
ALT	IU/L	16.1 ± 8.4	16.5 ± 8.1	0.65

## References

[1] Kuroda R., Kazumura K., Ushikata M., Minami Y., Kajiya K. (2018). Elucidating the improvement in vascular endothelial function from Sakurajima daikon and its mechanism of action: A comparative study with *Raphanus sativus*. J. Agri. Food Chem..

[2] Wakamatsu M., Yamanouchi H., Sahara H., Iwanaga T., Kuroda R., Yamamoto A., Minami Y., Sekijima M., Yamada K., Kajiya K. (2019). Catechin and caffeine contents in green tea at different harvest periods and their metabolism in miniature swine. Food Sci. Nutr..

[3] Kajiya K., Yamanouchi H., Tanaka Y., Hayashi H., Minami Y. (2020). Capsicum cultivated under adverse conditions produces high concentrations of antioxidants and capsaicinoids. J. Agri. Sci..

[4] Vanhoutte P.M., Shimokawa H., Feletou M., Tang E.H.C. (2018). Endothelial dysfunction and vascular disease—A 30th anniversary update. J. Agri. Food Chem..

[5] Bekendam R.H., Iyu D., Passam F., Stopa J.D., De Ceunynck K., Muse O., Bendapudi P.K., Garnier C.L., Gopal S., Crescence L. (2018). Protein disulfide isomerase regulation by nitric oxide maintains vascular quiescence and controls thrombus formation. J. Thromb. Haemost..

[6] Gliozzi M., Scicchitano M., Bosco F., Musolino V., Carresi C., Scarano F., Maiuolo J., Nucera S., Maretta A., Paone S. (2019). Modulation of Nitric Oxide Synthases by Oxidized LDLs: Role in Vascular Inflammation and Atherosclerosis Development. Int. J. Mol. Sci..

[7] Guinness World Records Heaviest Radish. http://www.guinnessworldrecords.com/world-records/heaviest-radish.

[8] American Chemical Society (2018). Compounds in ‘monster’ radish could help tame cardiovascular disease. J. Agric. Food Chem..

[9] Toshinari O., Sato H., Igarashi K. (2009). Anti-diabetic effects of pumpkin its components, trigonelline and nicotinic acid, on Goto-Kakizaki rats. Biosci. Biotechnol. Biochem..

[10] Zhou J., Chan L., Zhou S. (2012). Trigonelline: A plant alkaloid with therapeutic potential for diabetes and central nervous system disease. Curr. Med. Chem..

[11] Kano Y., Fukuoka N. (1996). Role of endogenous cytokinin in the development of hollowing in the root of Japanese radish (*Raphanus sativus* L.). Sci. Hortic..

[12] Kano Y., Fukuoka N. (1995). Effects of soil temperature on hollowness in Japanese radish (*Raphanus sativus* L. cv. ‘Gensuke’). Sci. Hortic..

[13] Thijssen D.H.J., Bruno R.M., van Mil A.C.C.M., Holder S.M., Faita F., Greyling A., Zock P.L., Taddei S., Deanfield J.E., Luscher T. (2019). Expert consensus and evidence-based recommendations for the assessment of flow-mediated dilation in humans. Eur. Heart J..

[14] Corretti M.C., Anderson T.J., Benjamin E.J., Celermajer D., Charbonneau F., Creager M.A., Deanfield J., Drexler H., Gerhard-Herman M., Herrington D. (2002). Guidelines for the ultrasound assessment of endothelial-dependent flow-mediated vasodilation of the brachial artery: A report of the International Brachial Artery Reactivity Task Force. J. Am. Coll. Cardiol..

[15] Neunteufl T., Katzenschlager R., Hassan A., Klaar U., Schwarzacher S., Glogar D., Bauer P., Weidinger F. (1997). Systemic endothelial dysfunction is related to the extent and severity of coronary artery disease. Atherosclerosis.

[16] Stennert A., Maier H.G. (1994). Trigonelline in coffee. II. Content of green, roasted and instant coffee. Zeitschrift fur Lebensmittel-Untersuchung und Forschung.

[17] Mizuno K., Matsuzaki M., Kanazawa S., Tokiwano T., Yoshizawa Y., Kato M. (2014). Conversion of nicotinic acid to trigonelline is catalyzed by *N*-methyltransferase belonged to motif *B*-methyltransferase family in *Coffea Arabica*. Biochem. Biophys. Res. Commun..

[18] Zheng X.Q., Matsui A., Ashihara H. (2008). Biosynthesis of trigonelline from nicotinate mononucleotide in mungbean seedlings. Phytochemistry.

[19] Zheng X.Q., Hayashibe E., Ashihara H. (2005). Changes in trigonelline (*N*-methylnicotinic acid) content and nicotinic acid metabolism during germination of mungbean (*Phaseolus aureus*) seeds. J. Exp. Bot..

[20] Nordestgaard B.G. (2016). Triglyceride-rich lipoproteins and atherosclerotic cardiovascular disease: New insights from epidemiology, genetics, and biology. Circ. Res..

[21] Anwar S., Bhandari U., Panda B.P., Dubey K., Khan W., Ahmad S. (2018). Trigonelline inhibits intestinal microbial metabolism of choline and its associated cardiovascular risk. J. Pharm. Biomed. Anal..

[22] Sharma L., Lone N.A., Knott R.M., Hassan A., Abdullah T. (2018). Trigonelline prevents high cholesterol and high fat diet induced hepatic lipid accumulation and lipo-toxicity in C57BL/6J mice, via restoration of hepatic autophagy. Food Chem. Toxicol..

[23] Liu L., Du X., Zhang Z., Zhou J. (2018). Trigonelline inhibits caspase 3 to protect β cells apoptosis in streptozotocin-induced type 1 diabetic mice. Eur. J. Pharmacol..

[24] Li Y., Li Q., Wang C., Lou Z., Li Q. (2019). Trigonelline reduced diabetic nephropathy and insulin resistance in type 2 diabetic rats through peroxisome proliferator-activated receptor-γ. Exp. Ther. Med..

[25] Van Dijk A.E., Olthof M.R., Meeuse J.C., Seebus E., Heine R.J., van Dam R.M. (2009). Acute effects of decaffeinated coffee and the major coffee components chlorogenic acid and trigonelline on glucose tolerance. Diabetes Care.

[26] Nathan J., Panjwani S., Mohan V., Joshi V., Thakurdesai P.A. (2014). Efficacy and safety of standardized extract of *Trigonella foenum-graecum* L. seeds as an adjuvant to L-Dopa in the management of patients with Parkinson’s disease. Phytother. Res..

[27] Bakuradze T., Lang R., Hofmann T., Eisenbrand G., Schipp D., Galan J., Richling E. (2015). Consumption of a dark roast coffee decreases the level of spontaneous DNA strand breaks: A randomized controlled trial. Eur. J. Nutr..

